# The Safety and Efficacy of Resolving Burr Entrapment by the Ping-Pong and Mother-in-Child Techniques

**DOI:** 10.7759/cureus.52893

**Published:** 2024-01-25

**Authors:** Cong Thanh Nguyen, Lam Truong Hoai, Duc Nguyen Hung, Minh Tran Duc

**Affiliations:** 1 Cardiovascular, Vietnam National Heart Institute, Hanoi, VNM; 2 Cardiology, Tam Anh Hospital, Hanoi, VNM

**Keywords:** coronary artery lesion (cal), rotablator, complication of treatment, coronary artery intervention, complex pci

## Abstract

Burr entrapment is a serious risk when performing rotational atherectomy on specific anatomical features of lesions such as tortuosity, calcification, and acute angulation. This occurrence, known as the *Kokeshi *phenomenon in Japanese, is caused by the burr’s proximal section being unable to ablate while pulling back the burr, leaving the distal end of the burr covered in diamond crumbs capable of lesion ablation following rotation. There are reports of different approaches used to retrieve an entrapped rotablator burr. In this case, we demonstrate that the ping-pong and mother-in-child techniques, which use separate guide catheters to engage the same coronary artery wiring across the lesion afterward and deep engagement of guide extension catheter manual traction, are highly effective and secure methods for retrieval.

## Introduction

As frequently demonstrated, rotational atherectomy (RA) offers better success rates when treating complex fibrocalcific lesions. In cases of potentially untreated, complicated lesions, RA often results in procedural success rates of over 90% and complication rates below 5% [1]. However, compared with routine balloon angioplasty and stenting, RA is a more technically demanding procedure with a higher risk of complications. This means that reducing RA problems entails a careful procedure. There exist complications specific to the rotablator system-such as burr entrapment and RotaWire fracturing, as well as complications solely associated with the RA procedure such as slow flow/no-reflow, bradycardia, and atrioventricular block. Entrapment of the rotablation burr or trapped rotablator are other terms for a stuck rotablator, which is one of the uncommon but potentially fatal consequences. The occurrence can be described in terms of the rotablation burr becoming trapped in a coronary lesion and being unable to be rotated or removed. Acute coronary obstruction from a stuck rotablator can occasionally require emergency cardiac surgery.

## Case presentation

**Figure 1:**
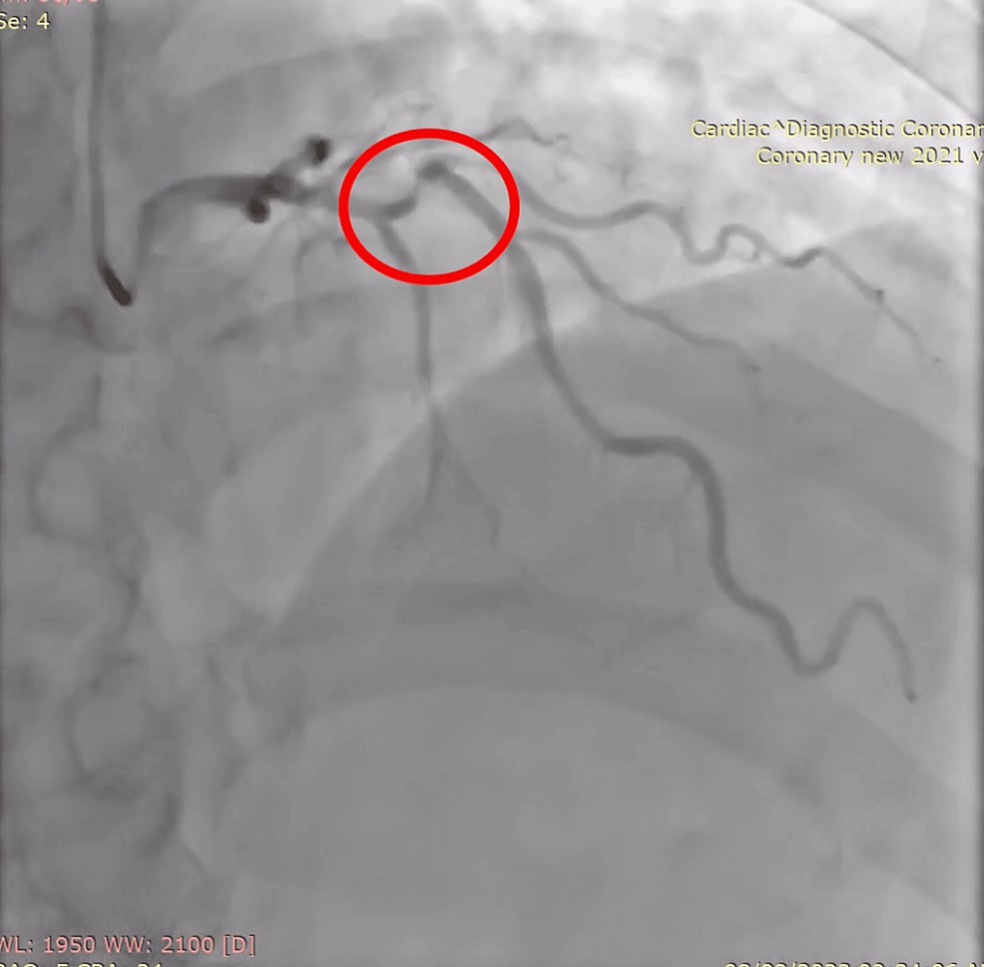
Angiography revealed acute angulation and highly calcified lesions were seen in the LAD (red circle) LAD: left anterior descending

A 74-year-old man was admitted to our institution with stable angina and a history of significant three-vessel coronary artery disease, diabetes, and hypertension. He had 90% stenosis of the mid-left anterior descending (LAD), a drug-coated stent in the proximal right coronary artery, and a chronic total occlusion of the left circumflex coronary artery. Acute angulation and highly calcified lesions were seen in the LAD during coronary angiography (Figure 1).

We decided to rotablate the LAD. Using trans-radial access, a 7 French EBU (Medtronic, Minneapolis, MN, USA) guiding catheter, and a runthrough wire (Terumo, Tokyo, Japan), we wired with some difficulty. To replace the runthrough wire, a RotaFloppy wire (Boston Scientific, Marlborough, MA, USA) was pushed into the distal LAD using a FineCross (Terumo). Rotablation began with using a 1.25 mm burr with a speed of 180,000 rpm. On the fifth pass, the burr was able to pass through the stenosis but could not be withdrawn from the LAD’s distal segment (Video 1).

**Video 1:** Fluorography showed the burr stuck at the site of the lesion

First, the rotablation system was gently pulled back by hand, but the attempt was proven to be ineffective. Moreover, extreme force induced perforation in the proximal LAD, but rapid echocardiography could not reveal pericardial effusion and hemodynamic stability (Video 2).

**Video 2:** Fluorography showed the Rota system was first gently pulled back by hand, but the attempt was proven to be ineffective

Next, we placed a second 6 French EBU 3.5 guiding catheter through the femoral artery, a process known as the ping-pong technique, and an antegrade runthrough wire across the lesion, which keeps the guidewire in the true lumen. Following this, we disconnected the rotablation system's drive sheath, slipped a Heartrail ST01 5Fr (Terumo) catheter through the remaining system, and then anchored the catheter tip by simultaneously pushing and pulling the rotablator. This was ultimately successful in removing the rotablation system (Video 3).

**Video 3:** Fluorography showed that this was successful in removing the rotablation system

Following a few procedures and patient stabilization, percutaneous coronary intervention was continued. The predilatation of the LAD lesion was attempted at 12 atm using a 2.0 × 15 mm IKAZUCHI (Kaneka, Osaka, Japan), and the 2.75 × 24 mm Resolute Onyx stent (Medtronic, USA) was delivered with ease as a result of adequate balloon preparation. Without pericardial effusion on echocardiography and hemodynamic stability, angiography revealed the existence of Ellis grade 1 proximal LAD (Video 4).

**Video 4:** Fluorography revealed the existence of perforation with Ellis grade 1

Nevertheless, we decided to cover the perforated location with a 3.0 × 15 mm PK Papyrus (Biotronik AG, Bülach, Switzerland). Finally, coronary angiography revealed TIMI 3 antegrade flow in the LAD. After the procedure, the follow-up was unremarkable, and the patient was discharged one day later.

## Discussion

The olive-shaped burr has a diamond coating on its distal surface for antegrade ablation; however, backward ablation is not permitted because the proximal part of the burr is smooth and devoid of diamonds. A burr may become entrapped if it advances beyond a severely calcified lesion or an angulated lesion that is extensively calcified. Previous proposals were made for these two mechanisms.

Initially, the small burr can be pushed through significantly calcified plaque before there is adequate ablation, particularly if the burr is being pushed forcefully and rapidly. The frictional heat during high-speed rotation may cause the space between the plaques to widen [2]. Conversely, the coefficient of friction is lower in motion than at rest, which would make it easier for the burr to pass through the calcified lesion without significantly debulking the calcified tissue.

Next, a highly calcified lengthy lesion, particularly one that is angulated and simultaneous with a coronary spasm, might include the burr. This kind of entrapment may happen if the burr were to push forcefully against the lesion without adequate pecking rotation. This would cause the rotational speed to decline rapidly [3].

Even though burr entrapment is an unusual side effect of rotablation, it is extremely challenging to retrieve a stuck burr without surgery. However, removal via surgery involves a lengthy, invasive procedure, especially in cases where the patient is hemodynamically compromised, and it is not always immediately possible. Before transporting the patient into the operating room for surgery, it may be worth trying several alternate options first to retrieve the lodged burr.

Manually withdrawing the rotablator system is the simplest method for dislodging the trapped burr. However, in this case, the stuck burr could not be withdrawn, and the vessel suffered from perforation and proximal segment injuries [4]. Previous studies have documented the removal of an entrapped rotablator burr utilizing a child-in-mother catheter. In this case, we used a Heartrail ST01 5Fr catheter for the first time to release the entrapped rotablator burr. Due to its monorail design, which makes it simple to deliver other devices, we were able to cut off the drive shaft and sheath near the advance and insert the Heartrail catheter into the entrapped burr via the remaining rotablation system. The catheter tip can act as a wedge between the burr and the surrounding plaque by simultaneously pushing on the burr shaft and counter-pulling on the Heartrail catheter. This potentially exerts a stronger and more direct pulling effort to remove the burr [5]. In so doing, the lesion's potential for major dissection or perforation after burr retrieval is avoided. We utilized the double-guiding catheter technique and advanced guidewire through another guiding catheter, which is the ping-pong technique reported by Silver et al. [6,7]. Moreover, following successful burr removal, the ping-pong and mother-in-child techniques may protect the proximal vessels and make subsequent interventional procedures more straightforward.

## Conclusions

The current situation suggests that a trapped rotablator burr could be dislodged using a Heartrail catheter. It is safer and more efficient to use the Heartrail catheter when combined with the double guide catheter approach. All things considered, interventional cardiologists who perform RA frequently can regard this procedure as fairly helpful.
